# Role of Platelet-activating factor and HO-1 in mediating the protective effect of rupatadine against 5-fluorouracil-induced hepatotoxicity in rats

**DOI:** 10.1007/s11356-022-18899-4

**Published:** 2022-02-04

**Authors:** Hanaa Mohamed Khalaf, Sara Mohamed Naguib Abdel Hafez, Ahlam Mohamed Abdalla, Nermeen N. Welson, Walaa Yehia Abdelzaher, Fatma Alzhraa Fouad Abdelbaky

**Affiliations:** 1 Department of Pharmacology, Faculty of Medicine, Minia University, Minia, Egypt; 2 Department of Histology and Cell Biology, Faculty of Medicine, Minia University, Minia, Egypt; 3 Department of Biochemistry, Faculty of Medicine, Minia University, Minia, Egypt; 4 Department of Forensic Medicine and Clinical Toxicology, Faculty of Medicine, Beni-Suef University, Beni-Suef, Egypt; 5 Department of Anatomy, Faculty of Medicine, Minia University, Minia, Egypt

**Keywords:** 5-Fluorouracil, Rupatadine, Hepatotoxicity, Caspase-3, iNOS

## Abstract

5-fluorouracil (5-FU) is a widely used chemotherapeutic drug, but its hepatotoxicity challenges its clinical use. Thus, searching for a hepatoprotective agent is highly required to prevent the accompanied hepatic hazards. The current study aimed to investigate the potential benefit and mechanisms of action of rupatadine (RU), a Platelet-activating factor (PAF) antagonist, in the prevention of 5-FU-related hepatotoxicity in rats. Hepatotoxicity was developed in male albino rats by a single 5-FU (150 mg/kg) intra-peritoneal injection on the 7th day of the experiment. RU (3 mg/kg/day) was orally administrated to the rodents for 10 days. Hepatic toxicity was assessed by measuring both liver and body weights, serum alanine aminotransferase and aspartate aminotransferase (ALT and AST), hepatic oxidative stress parameters (malondialdehyde (MDA), nitric oxide levels (NOx), reduced glutathione (GSH), superoxide dismutase (SOD)), and heme oxygenase-1 (HO-1). Inflammatory markers expressions (inducible nitric oxide synthase (iNOS), tumor necrosis factor-alpha (TNFα), interleukins; IL-1B, IL-6), the apoptotic marker (caspase-3), and PAF were measured in the hepatic tissue. 5-FU-induced hepatotoxicity was proved by the biochemical along with histopathological assessments. RU ameliorated 5-FU-induced liver damage as proved by the improved serum ALT, AST, and hepatic oxidative stress parameters, the attenuated expression of hepatic pro-inflammatory cytokines and PAF, and the up-regulation of HO-1. Therefore, it can be concluded that RU pretreatment exerted a hepatoprotective effect against 5-FU-induced liver damage through both its powerful anti-inflammatory, antioxidant, and anti-apoptotic effect.

## Introduction

Malignancy is the major cause of global death and no adequate therapeutic approaches are available. Chemotherapy is one of the most widely used agents to manage distinct types of cancer. Chemotherapeutic agents do not differentiate between normal and cancer cells leading to severe and undesirable toxicities. 5-FU, a pyrimidine analog anti-metabolite, is a common chemotherapeutic agent used for the treatment of different malignancies Al-Asmari et al. (2016).

After the introduction 5-FU as a chemotherapeutic drug, it has maintained a considerable clinical relevance. Certain tumor types, including colorectal, breast, esophageal, and head and neck tumors, are managed with 5-FU-based palliative chemotherapy Liu et al. (2017). An essential restriction of 5-FU use is its toxicity on normal growing body cells of various organs and tissues Al-Asmari et al. (2016). 5-FU cytotoxic effect on malignant and normal proliferating cells is owing to its catabolic pathway and the production of three main reactive metabolites; 5-fluorouridine- 50-monophosphate, 5-fluoro-uridine-50-triphosphate, and 5-fluoro-20-deoxyuridine-50-triphosphate. These metabolites exert their cytotoxic action via the perturbation of thymidylate synthase and the synthesis of nucleic acids. 5-FU toxicity is mainly dose-dependent, and it varies among the patients causing occasional therapy discontinuation (Garg et al. 2012; Rashid et al. 2014).

Common insufferable and serious 5-FU side effects include diarrhea, mucositis, dermatitis, myelosuppression, cardiotoxicity, genital organ toxicity, and hepatorenal toxicity. The chief mechanisms of 5-FU-induced cytotoxicity include reactive oxygen species overproduction and inflammatory mediators release that have the central effect in the 5-FU-induced toxicity Al-Asmari et al. (2016).

The impairment of hepatic function is a clinically important complication that frequently occurs in the patients treated with chemotherapy and it may require withdrawal of the administered drugs increasing the risk of treatment failure. Hence, the attempt to ameliorate the hepatic toxicity of 5-FU is an important step to improve its chemotherapeutic outcomes. Previous studies suggested that 5-FU caused oxidative stress in the liver and eventually resulted in structural and functional impairments of the hepatocytes (Ray et al. 2007; El-Hoseany 2012; Fukuno et al. 2016). The use of anti-inflammatory and/or antioxidant agents has been suggested to attenuate the chemotherapy-induced toxicity (Diba et al., 2021; Akindele et al. 2018). Several agents were used to ameliorate 5-FU-related toxicities on different organs without affecting its antineoplastic efficacy, but up till now, there is no consensus regarding the optimum drug therapy (Agbarya et al. 2014).

Platelet-activating factor (PAF) is a potent lipid mediator of different inflammatory processes that is synthesized by almost all cells. Its receptors are mainly present in the hepatocytes (Karantonis et al. 2010).

Rupatadine (RU) is a non-sedating H1-antihistaminic drug that was proved to have a PAF antagonistic activity. RU is commonly prescribed for allergic rhinitis, chronic urticaria, and diabetic nephropathy (Santamaria et al. 2018; Hafez et al. 2020). Picado (2006) reported that RU can suppress the degranulation of mast cells preventing the release of inflammatory mediators.

HO is expressed in three different isoforms: HO-1, 2, and 3. It is the rate-limiting enzyme in heme metabolism that catalyzes the degradation of pro-oxidant heme into carbon monoxide (CO), biliverdin, and ferrous ions and exhibits a wide range of cytoprotective anti-inflammatory, anti-apoptotic, and immunoregulatory effects in a variety of diseases (Pae et al. 2008; Hualin, 2012; Podkalicka et al. 2018). Several drugs produced a hepatoprotective effect in rats depending on HO-1 to exert antioxidant and anti-inflammatory actions (Aladaileh et al. 2019; Mingyang et al., 2020; Liu et al. 2020).

Also, the preparation and application of nanoparticles for different medical and pharmaceutical applications including the amelioration of toxicities were discussed before. Nanoparticles also seem to have promising therapeutic antitoxic effects (Amiri et al. 2019; Khoobi et al. 2019; Monsef et al., 2021).

Considering the deficient information about the pathophysiological function of PAF in the chemotherapy-associated hepatic damage, we evaluated for the first time the role of PAF in the pathogenesis of 5-FU-induced hepatotoxicity using RU as a PAF receptor antagonist. Currently, no reports are available about the protective effects of RU against the hepatotoxicity induced by 5-FU. Consequently, in the present study, we investigated RU as a potential hepatoprotective drug against 5-FU-induced hepatic damage in rats. We had a special focus on biochemical, histopathological, and immunological parameters. Also, we investigated the molecular mechanisms underlying RU’s action.

## Materials and Methods

### Chemicals

Rupatadine and 5-FU were attained from Sigma-Aldrich Co. (St. Louis, MO, USA). Urethane was purchased from CDH Fine Chemicals Co. (Darya Ganj*,* New Delhi*,* India). TNFα, IL-1B, and IL-6 kits were purchased from Invitrogen Thermo Fisher Scientific Inc./Lab Vision Co. (Fremont, CA, USA) (Catalog No: E-EL-R0019, and LOT 192,587,043, respectively). ALT and AST were purchased from Lab Dimension Co. (Cairo, Egypt). PAF kits were obtained from universal Biologicals Co. (Cambridge, United Kingdom). HO-1 ELISA kits were purchased from Assay Genie Co. (Dublin, Ireland) **(**Catalog No: SKU: RTFI00859). The ready-to-use iNOS and cleaved caspase-3 polyclonal rabbit antibodies were obtained from Thermo Fisher Scientific Inc./Lab Vision Co. (Fremont, CA, USA).

## Animals

The current experiment was conducted on Wistar male albino rats, 9–11 weeks old, weighing 200–250 g. They were obtained from the National Research Centre, Giza, Egypt, and were settled in cages (3 rats/cage) with free access to tap water and commercial rat chow diet (El- Nasr Co., Cairo, Egypt) at 24 ± 2 °C with a 12-h dark/light. The rodents were kept for two weeks before starting the study for acclimatization to the laboratory environment, and they were handled following ARRIVE ethical guidelines and the approval by the board of the Faculty of Medicine, Minia University, Egypt (239:7/2019).

## Experimental design

Animals were randomly arranged into four groups (8 rats/ group), as follows:

- **Group 1**: (the control group) received vehicle; 1 ml of 1% carboxymethyl cellulose (CMC), orally once daily for 10 days and single IP injection of 0.9% saline on the 7th day of the experiment.
- **Group 2**: (the RU group) received RU in a dose of 3 mg/kg/day orally once daily for 10 days, and single *IP* injection of 0.9% saline on the 7th day of the experiment, RU was suspended in 1% CMC (Hafez et al. 2020).
- **Group 3**: (the 5-FU group) received 1 ml of CMC orally once daily for 10 days and single IP injection of 5-FU (150 mg/kg) on the 7th day of the experiment (Al-Asmari et al. 2016).
- **Group 4**: (the 5-FU + RU group) received RU (3 mg/kg/day orally once daily for 10 days) and a single IP injection of 5-FU (150 mg/kg) on the 7th day.

In the present experiment, the oral drugs were administered once daily using an intragastric tube.

## Sample collection and storage

Twenty-four hours after the last dose (after the 10 days of the experiment), the animals were weighed and anesthetized with urethane (125 mg /kg, IP). Blood samples were collected from the abdominal aorta then centrifuged for 10 min at 4000 rpm to obtain clear serum. Collected sera were kept at –80 °C for biochemical assessments. The liver was excised and washed with saline. For each rat, the whole excised liver was weighed and divided into three parts. One part was kept in 10% formalin and embedded in paraffin for histopathological and immunohistochemical measurements, and another part was stored at –80 °C for real-time polymerase chain reaction (RT-PCR) testing. The remaining part of the liver was homogenized in ice-cold phosphate buffer (0.01 M, pH 7.4; 20% w/v) and centrifuged for 15 min at 4000 rpm, and then, the supernatant was kept at –80 °C for the different biochemical analyses.

## Biochemical measurements

### Measurement of serum ALT and AST

Serum ALT and AST levels were quantified using enzymatic kinetic kits and following the manufacturer’s recommendations (catalog NO: EZ016LQ for ALT and EZ012LQ, for AST).

## Measurement of hepatic oxidative stress parameters

Hepatic malondialdehyde (MDA) level was biochemically measured using the spectrophotometry method and depending on the reaction between MDA and the thiobarbituric acid as described by (Uchiyama and Mihara 1979). The colored complexes were estimated at 535 nm and then were calculated by using the standard curve of 1,1,3,3-tetra methoxy propane (Buege and Aust 1978).

Superoxide dismutase (SOD) activity was chemically evaluated as described by Marklund and Marklund (1974) who stated that SOD can inhibit the autoxidation of pyrogallol. One unit of SOD is equal to the percent of enzyme which attenuates pyrogallol autoxidation by 50%. Hepatic tissue activity of SOD was spectrophotometrically estimated at 420 nm.

Reduced glutathione (GSH) activity was biochemically evaluated in the hepatic tissue homogenates as described previously (Moron et al. 1979). The GSH evaluation method depends on the reduction of Ellman's reagent by the thiol groups of GSH to a yellow colored 5-thio-2-ni-trobenzoic acid that is detected at 412 nm using the Beckman DU-64 UV/VIS spectrophotometer.

The total nitrite/nitrate (NOx), the stable oxidation end product of nitric oxide, was quantified in the tissue homogenates using the Griess reaction that depends on the interaction of nitrite with the blend of naphthyl ethylene diamine and sulfanilamide. NOx level was estimated at 540 nm using the Beckman DU-64 UV/VIS spectrophotometer (Sastry et al. 2002).

## Evaluation of the hepatic level of inflammatory parameters

The hepatic tissue levels of TNF-ɑ, IL-1β, and IL-6 were measured using their ELISA kits; Invitrogen Thermo Fisher Scientific Inc./Lab Vision Co. (Fremont, CA, USA) (Catalog No: E-EL-R0019, and LOT 192,587,043, respectively) and following the manufacturer's instructions.

## Evaluation of the hepatic HO-1 level

The hepatic HO-1 level was detected using the ELISA kits (Assay Genie Co., Dublin, Ireland) **(**Catalog No: SKU: RTFI00859) and following the manufacturer's instructions.

## Evaluation of the hepatic PAF level

The hepatic PAF level was estimated by using the ELISA kits (Universal Biologicals Co., Cambridge, United Kingdom) (Catalog No**: **ER1226) and following the manufacturer's instructions.

## Real-time reverse transcription-polymerase chain reaction (RT-PCR)

The total RNA was extracted from the hepatic tissues using the RiboZol reagent (Amresco, Solon, USA). 5 µg of the total RNA was used for RT-PCR following the manufacturer's instructions (Thermo Scientific Verso sybr green one-step qRT-PCR kits plus ROX Vial, code no AB-4104/A) in the thermal cycler (Applied Biosyst 7500 fast, Techne (Cambridge) LTD., UK). RT-PCR cycling parameters were kept at 50 °C for 15 min., 95 °C for 15 min and 40 cycles of denaturation at 95 °C for 15 s, annealing at 60 °C for 30 s, and then extension at 72° C for 30 s. The total RNA purity was determined through the absorption ratio 260/280 nm. It was about 1.8–2 for all the preparations. The relative gene expression of HO-1 was calculated using the comparative threshold cycle method (Ct). All the values were normalized to the β-actin gene. The used sets of primers were:

**HO-1** **primer sequence (**5′-3′**).**

Forward primer: 5′- TTAAGCTGGTGATGGCCTCC -3′

Reverse primer: 5′- GTGGGGCATAGACTGGGTTC-3′

**β-actin primer sequence (**5′-3′**).**

Forward: 5'-GTCGTACCACTGGCATTGTG-3'.

Reverse: 5'-CAGCATGGTGACCGTAACA-3'.

## Histopathological and immunohistochemical study

At the end of the experiment, fresh small pieces of the left hepatic lobe were excised from each animal, rapidly fixed in 10% neutral-buffered formalin, dehydrated in a graded alcohol series, washed with xylene, and embedded in paraffin wax. The Sects. (5 μm thickness) were stained with the hematoxylin and eosin stain (H&E) for studying the general histological architecture (Morris et al. 2019). Other slides were stained with Masson’s trichrome for the collagen fiber detection (Suvarna et al. 2018). Additional slides were preceded for the immunohistochemical study using rabbit monoclonal antibodies. The first one was the anti-inducible nitric oxide synthase (iNOS) antibodies [(diluted at 1: 500); catalog number is ab178945] that are produced recombinantly (animal-free) for high batch-to-batch consistency and long-term security of supply, suitable for ELISA, WB, ICC/IF, and IP, reacts with mice, rats, and humans, expressed in the liver, retina, bone cells, and airway epithelial cells of the lung, and not expressed in the platelets. The second one was the anti-cleaved caspase-3 antibodies [ (diluted at 1: 1000), catalog NO: ab184787] that are produced recombinantly (animal-free) for high batch-to-batch consistency and long-term security of supply, suitable for WB, IHC-P, and IP, knockout validated, reacts with mouse, rat, and human, highly expressed in the lung, spleen, heart, liver, kidney, and the cells of the immune system, with moderate levels in the brain and skeletal muscle, and with low levels in the testis. The steps were done according to the manufacturer’s recommendations (Côté 1993).

## Photography

In this study, the camera (Olympus C-35DA-2, Japan) was attached to the microscope (Olympus CX23LEDRFS1, Olympus, Tokyo, Japan) and used at the Histology Department, Faculty of Medicine, Minia University.

## Morphometric study

Morphometric estimation using the Leica QWin 500 image analysis software (Leica Microsystems, Wetzlar, Germany) was performed to assess the cleaved caspase-3, and the iNOS immune-marked cells were counted in 10 adjacent non-overlapping fields of three liver sections of each rat.

## Statistical analysis

The statistics were displayed as means ± standard error of the mean (SEM). The results were analyzed using the one-way analysis of variance (ANOVA) test and the Tukey multiple comparison test. Statistical calculations were performed using GraphPad Prism-5 for Windows (Version 5.01, San Diego, California, USA). The significant difference was set at *p*-value < 0.05.

## Results

### Influence of RU on the body weight and liver index in 5-FU-induced hepatotoxicity

There was a significant decrease in the body weight and liver index (liver weight/body weight) in the 5-FU group as compared to the control group. Rodents pretreated with RU (the RU + 5-FU group) displayed a significant increase in their body weight and liver index as compared to the 5-FU group (Table 1). There was no significant difference in the serum activities of ALT and AST between the RU group and the control group.

**Table 1:** Effect of RU on the liver index and liver function enzymes (AST and ALT)

Group	ALT(U/L)	AST(U/L)	Liver index
Control	23.13 ± 1.61	20.40 ± 1.12	3.28 ± 0.19
RU	23.25 ± 2.18^b^	22.60 ± 2.04^b^	3.34 ± 0.15^b^
5-FU	61.88 ± 2.44^a^	60.60 ± 3.21^a^	1.52 ± 0.24^a^
5-FU + RU	41.38 ± 2.17^b^	41.40 ± 3.20^b^	3.17 ± 0.28^b^
F (df)	76.29 (3)	54.12 (3)	15.22 (3)
***P*** value	< 0.0001	0.0010	0.0069

Values are displayed as the mean ± SEM (n = 8). ^a^Significant difference from the control, ^b^Significant difference from the 5-FU group. RU: rupatadine; 5-FU: 5-fluorouracil, AST: aspartate aminotransferase, ALT: alanine aminotransferase, and df: degree of freedom.

## Influence of RU on liver enzymes in 5-FU-induced hepatotoxicity

The 5-FU group had a significant elevation in the levels of AST and ALT relative to the control group. RU pretreatment in the RU + 5-FU group significantly reduced the AST and ALT levels as compared to the 5-FU group (Table 1).

## Influence of RU on hepatic oxidative stress parameters in 5-FU-induced hepatotoxicity

As shown in Table 2, a significant reduction in the hepatic levels of SOD and GSH and significant elevations of MDA and NOx levels were observed in the rats treated with 5-FU alone as compared to the control group. There was a significant improvement in the oxidative stress parameters with RU pretreatment (RU + 5-FU group) in comparison with the 5-FU group as indicated by the significant increase in SOD and GSH along with the reduction of MDA and NOx levels (Table 2). There was no significant difference between the RU group and the control group.

**Table 2:** Influence of RU on hepatic oxidative stress parameters (GSH, SOD, MDA, and NOx)

Group	GSH (mg/g tissue)	SOD (U/g tissue)	MDA (nmol/g tissue)	NOx (nmol/g tissue)
Control	1.12 ± 0.15	1.49 ± 0.04	14.84 ± 0.73	0.12 ± 0.01
RU	1.20 ± 0.10^b^	1.43 ± 0.05^b^	14.61 ± 0.80^b^	0.11 ± 0.01^b^
5-FU	0.42 ± 0.02^a^	0.50 ± 0.03^a^	24.96 ± 1.60^a^	0.51 ± 0.02 ^a^
5-FU + RU	1.21 ± 0.11^b^	1.65 ± 0.05^b^	14.64 ± 1.25^b^	0.29 ± 0.02^b^
F (df)	10.84 (3)	116.2 (3)	19.67 (3)	97.64 (3)
*P* value	0.0004	0.0008	0.0005	< 0.0001

Values are shown as the mean ± SEM (n = 8). ^a^Significant variance from the control group, ^b^significant variance from the 5-FU group. RU: rupatadine, 5-FU:5-fluorouracil, MDA: malondialdehyde, SOD: superoxide dismutase, GSH: reduced glutathione, NOx: total nitrite/nitrate, and df: degree of freedom.

## Effect of RU on hepatic inflammatory parameters in 5-FU-induced hepatotoxicity

5‐FU significantly elevated the hepatic TNF-α, IL-1 β, and IL-6 levels in comparison with the control group. Meanwhile, RU pretreatment in the RU + 5-FU group significantly decreased the hepatic inflammatory parameters as compared to the 5-FU group**.** There was no significant difference between the RU group and the control group (Table 3).

**Table 3:** Effect of RU on hepatic PAF and inflammatory parameters (IL-1 β, IL-6, and TNF- ɑ)

**Group **	**TNF- ɑ(pg/g tissue)**	**IL-6(pg/g tissue)**	**IL-1**β**(pg/g tissue)**	**PAF(mg/g tissue)**
Control	2117 ± 80.9	167.1 ± 13.16	13.61 ± 0.74	0.78 ± 0.06
RU	2164 ± 108^b^	202.2 ± 11.65^b^	12.68 ± 0.85^b^	0.74 ± 0.08^b^
5-FU	3702 ± 185^a^	525.5 ± 26.46^a^	64.99 ± 4.24^a^	1.90 ± 0.07^a^
5-FU + RU	2326 ± 150^b^	193.7 ± 8.12^b^	15.65 ± 0.50^b^	0.90 ± 0.08^b^
F (df)	30.28 (3)	107.0 (3)	133.2 (3)	52.09 (3)
***P*** value	0.0046	0.0003	0.0001	0.0003

Values are displayed as the mean ± SEM (n = 8). ^a^Significant difference from the control, ^b^significant difference from the 5-FU group. RU: rupatadine, 5-FU: 5-fluorouracil, PAF: platelet-activating factor, TNF-α: tumor necrosis factor-alpha, IL: interleukin, and df: degree of freedom.

## Influence of RU on hepatic PAF level in 5-FU-induced hepatotoxicity

There was a significant increase in the hepatic level of PAF in the 5-FU group in comparison to the control group. Besides, RU pretreatment in the RU + 5-FU group significantly reduced the hepatic level of PAF in comparison with the 5-FU group. There was no significant difference between the RU group and the control group (Table 3).

## Influence of RU on the mRNA expression of HO-1 in 5-FU-induced hepatotoxicity

**Fig. 1:**
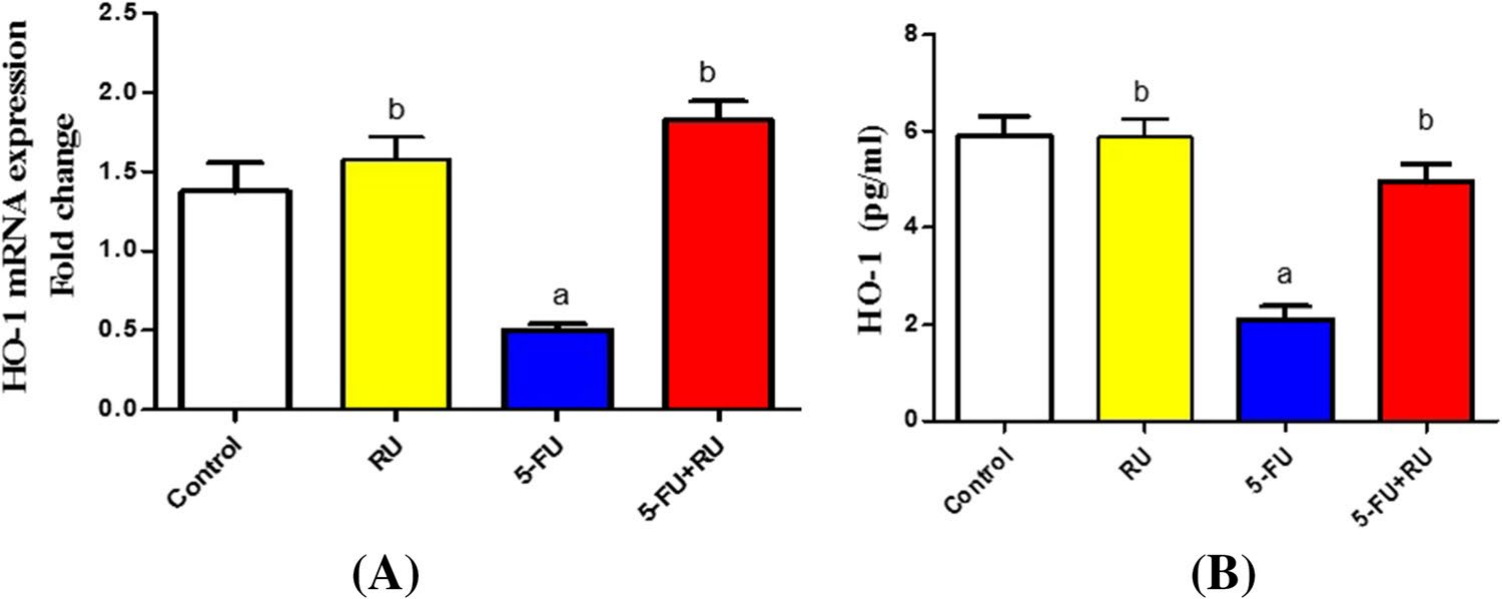
**Effect of RU on the mRNA expression of HO-1 in 5-FU-induced hepatotoxicity.** Data are displayed as the mean ± SEM (n = 8). ^a^Significant difference from the control, ^b^significant difference from the 5-FU group. RU: rupatadine, 5-FU: 5-fluorouracil, HO-1: heme oxygenase-1. *p* value = 0.0110, F (df):19.36 for the figure A and *p* value: < 0.0001, F (df): 24.38 for the figure B, respectively

Figure 1A presents a significant reduction in the hepatic HO-1 level of the 5-FU group as compared to the control group. In addition, rats that received RU pretreatment presented a significant elevation in the hepatic HO-1 level when compared with the 5‐FU group (Fig. 1B).

## Histopathological examination of the liver tissue in 5-FU-induced hepatotoxicity

**Fig. 2:**
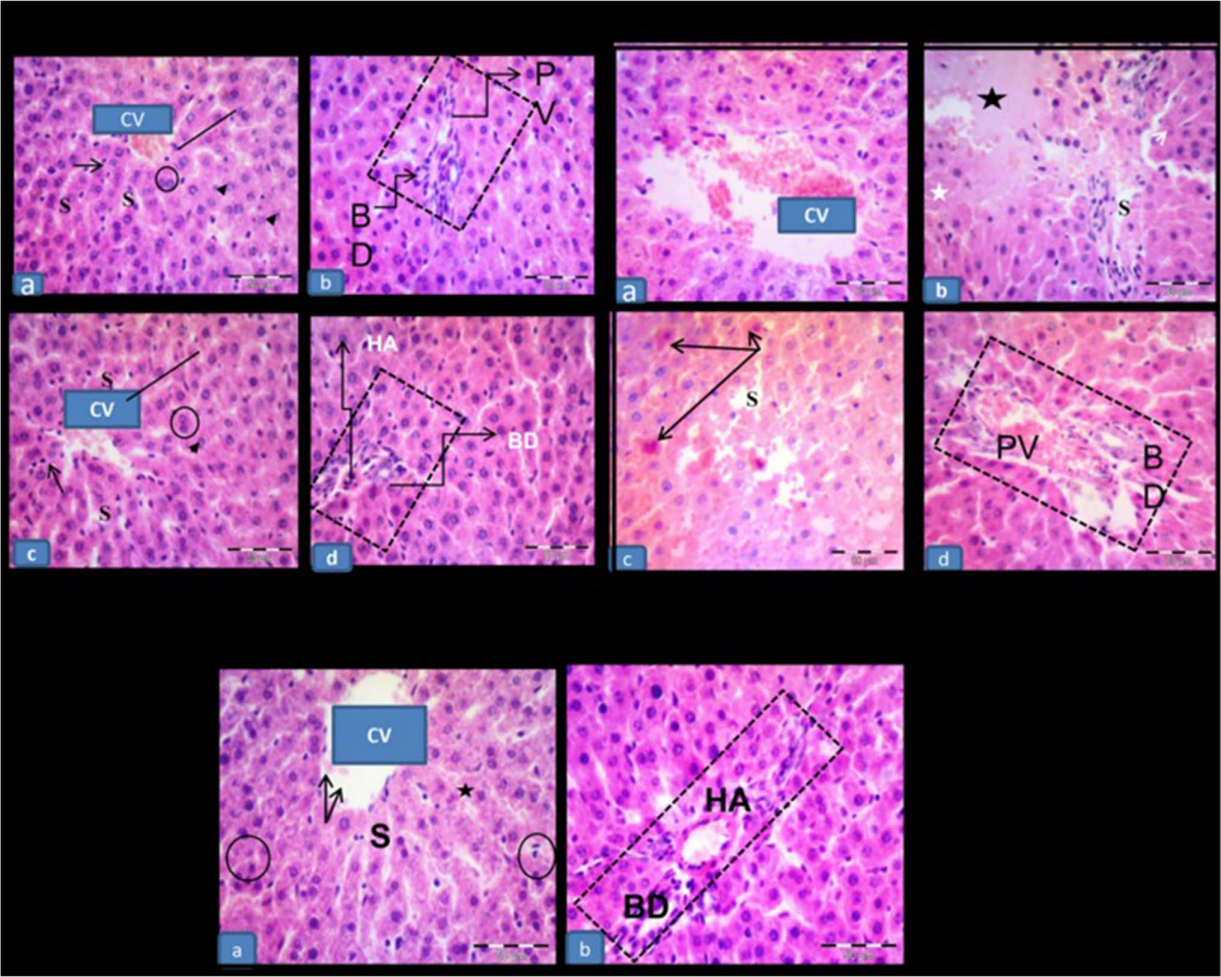
**Histopathological examination of the liver tissue in 5-FU-induced hepatotoxicity by H&E** Showing: **A)**:**(a,b)** the **control** and **(c,d) the RU group,** respectively. **(a,c)** Normal lobular architecture with numerous plates (lines) of polygonal acidophilic hepatocytes with rounded vesicular nuclei (arrows) is seen. Notice the hepatocytes radiating from the central veins (CV) and separated by blood sinusoids (S). Some hepatocytes showing binucleated nucleoli (circles). Kupffer cells (arrowheads) showing hanging in the blood sinusoids. **(b,d)** Portal tracts (rectangles) showing branches of the portal vein (PV), hepatic artery (HA), and bile duct (BD). **B): The 5-FU group** showing: **(a)** dilated congested central vein (CV). Most hepatic cells showing dark nucleoli. **(b)** Loss of normal lobular architecture (star). Note the degenerated hepatocytes with ghosts of nuclei (white arrow) and focal area of lytic necrosis (white star). **(c)** Single cell necrosis of hepatocytes (arrows) and dilated blood sinusoids (s). **(d)** Rectangle showing distorted portal area containing apparent dilatation of bile duct (Bd) and portal vein (PV). **C) The 5-FU + RU group s**howing: **(a)** Restored lobular architecture with mild central vein (CV) congestion and scattered areas of degenerations (star). Most liver cells show normal appearance and are arranged in cords (arrows). Less dilated blood sinusoids (s) and few cells with dense nuclei and deeply acidophilic cytoplasm (circle). **(b)** Portal area (rectangle) with less dilated hepatic artery (HA) and bile duct (BD). H&E, × 400; scale bar = 50 $\mu$ m

The control and RU groups displayed a normal lobular architecture. Numerous plates of hepatocytes radiating from the central veins and separated by blood sinusoids were observed. The hepatocytes seemed polyhedral with acidophilic cytoplasm, large central vesicular nuclei, and prominent nucleoli. Some hepatocytes were seen bi-nucleated. Kupffer cells were shown hanging in the blood sinusoids. The portal tracts exhibited branches of the portal veins, hepatic arteries, and bile ducts (Fig. 2A).

The 5-FU group showed a loss of the normal lobular architecture. Dilated congested central veins and blood sinusoids were frequently seen. Degenerated liver cells with ghosts of nuclei and focal area of lytic necrosis were noticed. Single-cell necrosis of the hepatocytes was also present. Regarding the portal areas, an apparent dilatation of the bile ducts and portal veins was noticed (Fig. 2B).

In contrast, the 5-FU + RU group showed an obvious morphological restoration of the normal lobular architecture but with few scattered areas of degenerations and less frequent central veins congestion when compared to the previous group. Almost all the hepatocytes had a normal appearance. Meanwhile, few dilated blood sinusoids and cells with dense nuclei and deeply acidophilic cytoplasm were still seen. The portal areas appeared with less dilated hepatic arteries and bile ducts compared to the 5-FU group (Fig. 2C).

## Effect of RU on the hepatic sections stained with the Masson's trichrome stain in 5-FU-induced hepatotoxicity

**Fig. 3:**
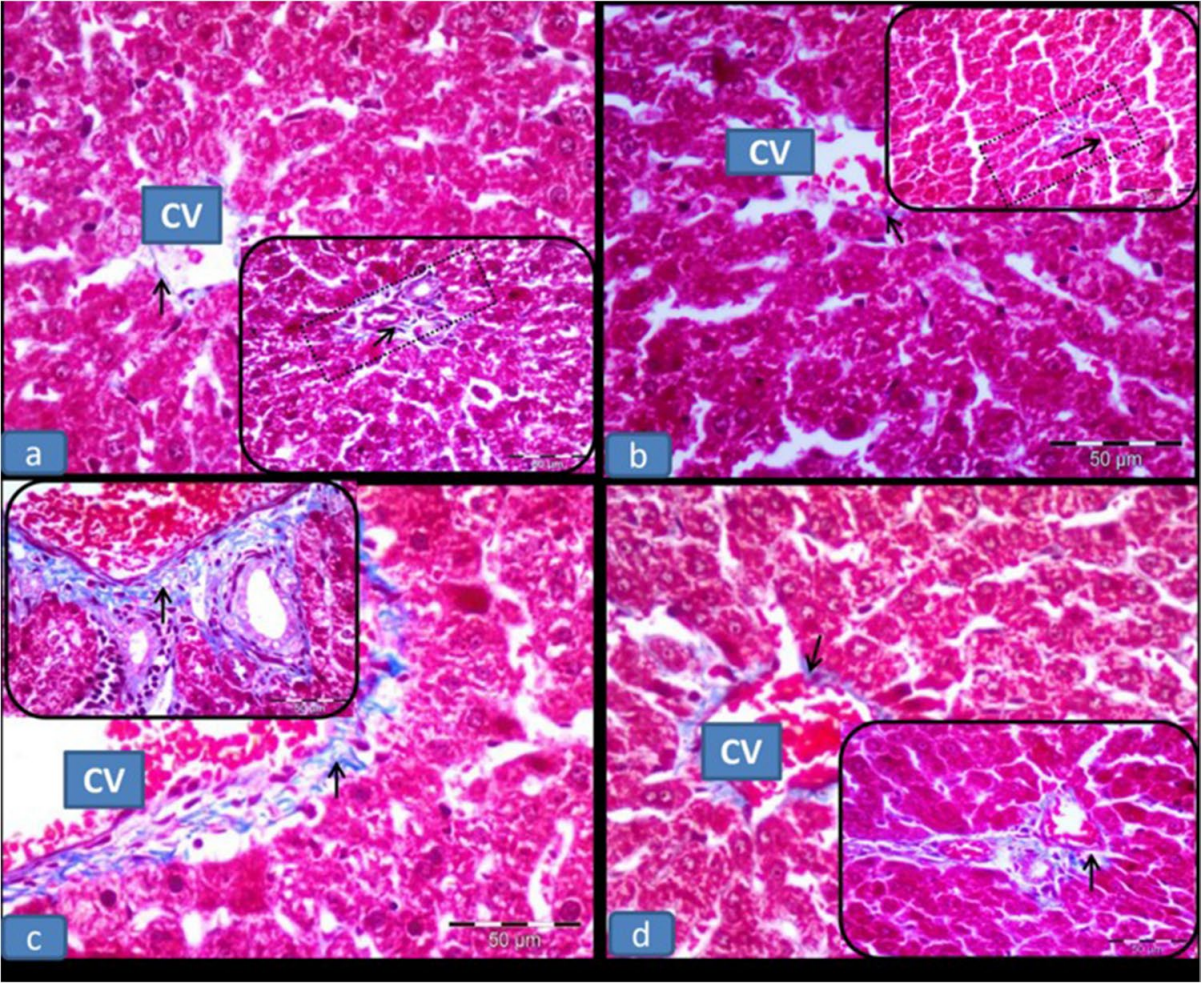
**Effect of RU on the hepatic sections stained with the Masson's trichrome stain in 5-FU-induced hepatotoxicity** Showing; **(a,b)** the control and RU groups, respectively, displaying scant collagen fibers (arrow) around the central vein (CV) and portal area (rectangle). **(c)** The 5-FU group showing more collagen fibers (arrow) surrounding the central vein (CV) and portal area (rectangle). **(d)** The 5-FU + RU group displaying little collagen fibers (arrow) around the previously mentioned areas. Masson’s trichrome × 400; scale bar = 50 $\mu$ m

As shown in Fig. 3, the control and RU groups showed scant collagen fibers surrounding the central veins and portal areas, while the 5-FU group exhibited more collagen fibers surrounding the previously mentioned areas. The 5-FU + RU group showed little collagen fibers surrounding the central veins and portal areas as compared to the 5-FU group.

## Immunohistochemical results of iNOS immunoexpression

**Fig. 4:**
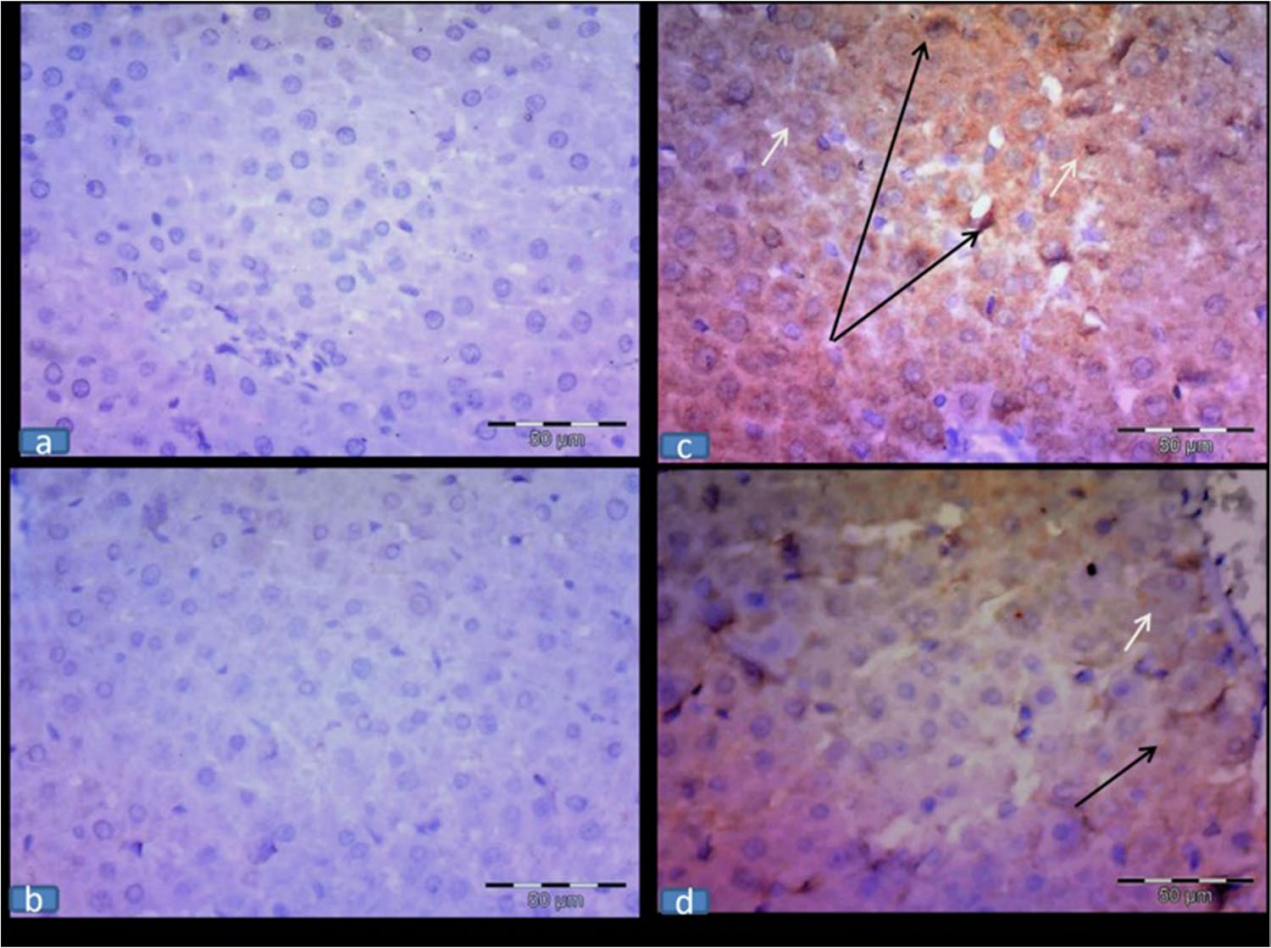
**Effect of RU on the immunohistochemical results of iNOS immunoexpression in 5-FU-induced hepatotoxicity (a,b)** The control and RU groups, respectively, showing a negative iNOS expression. **(c)** The 5-FU group showing a marked immunolabeling of the hepatocytes (white arrows) and von Kupffer cells (black arrows); (cytoplasmic expression). **(d)** The 5-FU + RU group displaying a faint immunolabeling of the previously mentioned areas. Immunohistochemistry, counterstained with H X400**;** scale bar = 50 $\mu$ m

The assessment of wide fields of the control and RU groups exhibited no iNOS immunolabeled cells, while the 5-FU group showed an intense immunolabeling of hepatocytes and von Kupffer cells (cytoplasmic expression). In contrast, the 5-FU + RU group showed a faint immunolabeling of the previously mentioned cells compared to the 5-FU group (Fig. 4).

## Immunohistochemical results of activated caspase-3 immunoexpression

**Fig. 5:**
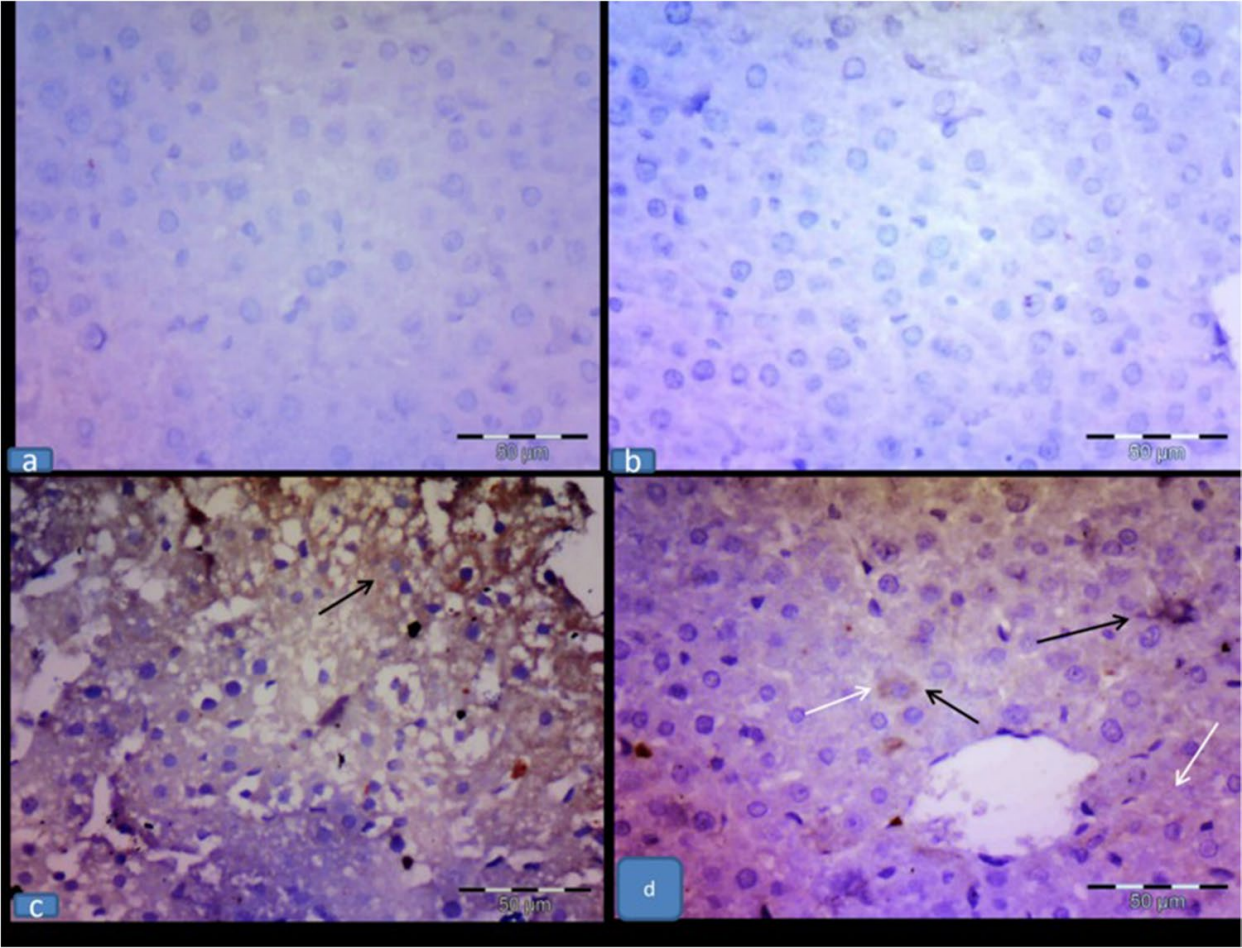
**Effect of RU on the immunohistochemical results of caspase-3 immunoexpression in 5-FU-induced hepatotoxicity** (**a&b**) The control and RU groups, respectively, showing a negative caspase-3 expression. (**c)** The 5-FU group displaying a marked immunolabeling of the hepatocytes (white arrows) and von Kupffer cells (black arrows); (cytoplasmic expression). **(d)** The 5-FU + RU group showing a faint immunolabeling of the hepatocytes (white arrows) but still few von Kupffer cells (black arrows) showing a strong expression. Immunohistochemistry, counterstained with H X400**;** scale bar = 50 $\mu$ m

The control and RU groups exhibited a negative caspase-3 expression. Meanwhile, the 5-FU group showed an intense cytoplasmic immunolabeling of the hepatocytes and von Kupffer cells. In contrast, the 5-FU + RU group displayed a scattered faint immunolabeling of the previously mentioned cells (Fig. 5).

## Morphometric results

A significant elevation of iNOS and caspase-3-immune-labeled hepatocytes and von Kupffer cells was observed in the 5-FU group compared to the control group. Meanwhile, a significant reduction was detected in the 5-FU + RU group compared to the 5-FU group (Table 4).

**Table 4:** Effect of RU on the mean number of cleaved caspase-3 and iNOS immune-labeled cells

Groups	Caspase-3-immune-labeled hepatocytes	Caspase-3-immune-labeled Kupffer cells	iNOS-immune-labeled hepatocytes	iNOS-immune-labeled Kupffer cells
Control	0.66 ± 0.21	0.33 ± 0.21	0.66 ± 0.21	0.33 ± 0.21
RU	0.83 ± 0.16 ^b^	0.83 ± 0.16^b^	0.8333 ± 0.16^b^	0.50 ± 0.22^b^
5-FU	47.50 ± 0.99^a^	11.16 ± 0.83^a^	27.83 ± 0.60^a^	14.83 ± 0.94^a^
5-FU + RU	19.00 ± 1.06 ^b^	3.33 ± 0.42^b^	7.66 ± 0.71^b^	5.50 ± 0.61^b^
F (df)	208.9 (3)	106.2 (3)	695.1 (3)	134.7 (3)
*P* value	0.0001	0.0001	0.0001	0.0002

Values are displayed as the mean ± SEM (n = 8). ^a^Significant variance from the control, ^b^significant variance from the 5-FU group. RU: rupatadine, 5-FU: 5-fluorouracil, and df: degree of freedom.

## Discussion

5-FU-induced toxicity was proven in animal models, and the pathogenetic mechanisms of this toxicity have been examined in different organs such as the kidneys and intestine** (**Rashid et al. 2014; Zhao et al. 2014; Mahmoud et al. 2020). In the current research, the liver damage was induced by 5-FU in male Wistar rats and RU was found to be a hepatoprotective agent against this toxicity. The RU hepatoprotective effect was indicated by biochemical, immunohistochemical, and histological assessments.

Liver injury could change the membrane's permeability leading to the increased release of some liver-specific enzymes, mainly AST and ALT (Rahim et al. 2014). Liver function tests are important for the assessment of hepatotoxicity; thus, an increase in the serum levels of AST and ALT indicates their leakage from the injured necrotic hepatocytes into the systemic circulation (Ozer et al. 2008). ALT and AST are essential biological markers for the cellular damage and toxicity, and they are used as an indicator of acute liver toxicity (Zeashan et al. 2009).

Marked oxidative production with the accumulation of oxidative radicles in the liver damaged the cellular membranes and the hepatic endothelial lining in the present study. Different forms of hepatic insufficiency in the 5-FU group were manifested by the significant elevations of AST and ALT, alterations of body weight and liver index, hepatic inflammation, oxidative damage, and hepatic cell apoptosis. These results were discordant with previous literature (Al-Asmari et al. 2016; Gelen et al. 2018).

The current histological findings went in line with the biochemical results. Various morphological changes were demonstrated in the 5-FU group in the form of distorted lobar architecture, degenerated hepatocytes, dilated central veins, and distorted portal areas. These results were in agreement with Alessandrino et al. 2019 who reported the 5‐FU-induced hepatotoxicity in the patients with colorectal cancer. Additionally, El-Sayyad et al. 2009 and Assayaghi  et al. 2019 stated that 5-FU markedly affected the histological structure of the liver.

In an attempt to explain the effect of 5-FU on accelerating the collagen fiber deposition, the Masson's trichrome stain was used to detect the collagen formation (Elbassuoni and SM, 2019; Hafez 2019). The 5-FU group showed more extensive collagen fibers surrounding the central veins and portal areas as compared to the normal rats. This finding was also reported by Bano and Najam 2019 who suggested that 5-FU induced direct hepatic toxicity, inflammation, necrosis, and collagenous fibril formation.

Shreds of evidence have demonstrated that oxidative stress mediates the 5-FU-induced organ toxicities (Rashid et al. 2014; Guo et al. 2015). Reactive oxygen species (ROS) generation causes the peroxidation of membrane lipids and oxidative cellular injury. Cellular antioxidant enzymatic and non-enzymatic defenses diminish the resultant tissue destruction (Polat Köse et al. 2015). ROS lead to cellular injury and necrosis in various tissues including the liver, kidney, and intestines. ROS damage also raises the portal and systemic levels of endotoxins and their hepatic translocation leading to neutrophil ingestion and higher levels of ROS release (Godos et al. 2017). The termination of ROS damage in healthy cells is complemented by the radical scavenging system including superoxide dismutase (SOD) and reduced GSH (Gulcin 2006).

Our results revealed that there was a significant reduction in the activity of the antioxidant enzymes in liver including SOD and GSH following the administration of 5-FU along with the increase in the hepatic MDA and NOx levels. These findings coincide with other previous reports (Arab et al. 2018; Gelen et al. 2018).

Several researchers have suggested that ROS are involved in the stimulation of extracellular signal-regulated kinase (ERK). The activation of the ERK pathway promotes transcription factors as the nuclear factor kappa B (NF-κB) which regulates the expression of various pro-inflammatory mediators (Arab et al. 2018). The role of the pro-inflammatory cytokines in the pathogenesis of hepatic toxicity and the cellular signaling pathways is still being researched (Laverty et al. 2010). It has been stated that these pro-inflammatory cytokines are related to a significant rise in serum IL-1ß, IL-6, and TNF-α levels following the 5-FU administration in rats (Chang et al. 2017).

Matching other earlier researches of Chang et al. 2017 and Arab et al. 2018, 5-FU administration in the present study resulted in a significant increase in the hepatic levels of the pro-inflammatory cytokines IL-1ß, IL-6, and TNF-α. Besides, the ROS produced by iNOS led to tissue injury. The 5-FU-associated increase in iNOS activity indicated the important role of ROS in the pathogenesis of 5-FU-induced tissue damage.

Our study revealed that cytoplasmic iNOS expression was prevalent in the hepatic and von Kupffer cells of the 5-FU group. Matching these findings, Leitão et al. 2007 reported that the increased iNOS activity leads to mucosal injury. El-Sayyad et al. 2009 also found that 5-FU initiates inflammatory cells infiltrations with a subsequent increase in iNOS expression.

In this current work, the cytoplasmic cleaved caspase-3 expression was enhanced in the hepatic and von Kupffer cells of the 5-FU group. Caspase-3 is an important regulator of apoptosis that becomes activated in the intrinsic and extrinsic apoptotic pathways in response to cytochrome C leakage from the mitochondria (Abdel-Aziz and Hafez 2019; Ahmed et al. 2019; Ponce-Cusi and Calaf 2016). The overexpression of caspase-3 in this experiment confirmed the damage of liver parenchyma and hepatic dysfunction in the 5-FU group.

In the present study, 5-FU administration significantly decreased the hepatic level of the cytoprotective HO-1 protein. However, it is worthy to note that the RU administration induced its expression in the hepatic tissue. These results suggest that RU ameliorates the oxidative stress-associated liver toxicity through the potentiation of cellular antioxidant defenses by stimulating HO-1 expression. These findings are in agreement with previous studies that reported the increased HO-1expression to reduce the neutrophils infiltration from bone marrow and function. Thus, it played an important anti-inflammatory and anti-apoptotic role in the liver damage models (Hyvelin 2010; Zhang et al. 2017).

PAF is a pro-inflammatory phospholipid mediator that is created by almost all cells and it is an efficient mediator in numerous inflammatory responses, induction of apoptosis, and NF-kB activation (Ahmed et al. 2021; Lu et al. 2008). PAF is synthesized by the activated polymorphonuclear and endothelial cells, and it is implicated in other pathological and physiological processes such as wound healing and angiogenesis (Stafforini et al. 2003). Additionally, it was documented that PAF is implicated in the pathogenesis of liver injury (Grypioti et al. 2005). Interestingly, the role of PAF in 5-FU-associated intestinal mucositis was studied in rats using a PAF receptor blocker and the production of PAF was involved in the pathogenesis of mucositis (Soares et al. 2011).

RU, which competitively blocks both histamine and PAF receptors, is well tolerated with a good safety profile in many clinical trials (Katiyar and Prakash 2009; Nettis et al. 2013). RU was reported to protect against pulmonary fibrosis by inhibiting the PAF-mediated pathway as it decreased the lung-infiltrating inflammatory cells, expression of the inflammatory cytokines, and mast cell degranulation in the injured lungs (Lv et al. 2013, Vasiadi et al. 2010).

In the current research, RU reversed the 5‐FU-associated hepatic toxicity owing to its blocking effect on the PAF receptors. The RU-pretreated rats showed a significant enhancement in their body weight, liver index, and liver function in comparison with the 5-FU group along with the improved anti-oxidative capacity indicated via the elevated hepatic levels of GSH, HO-1, and SOD activities and the decreased MDA and NO levels. Inhibited membrane lipid peroxidation and nitric oxide synthesis attenuated the level of the pro-inflammatory cytokine (TNF-ɑ, IL-1ß, and IL-6) in the hepatic tissue with the down-regulation of HO-1-mediated hepatic inflammation and apoptosis. These findings were accompanied by the restoration of normal histological patterns of the hepatic tissue as compared to the 5‐FU group. In accordance with these results, previous studies reported the protective effect of RU on different animal models (Hafez et al. 2020; Mohamed et al. 2021).

RU also attenuated iNOS and activated caspase-3 immunoexpression in the hepatic tissue and produced anti-inflammatory and anti-apoptotic actions as proven by the significant decrease in IL-6,1ß and TNF-α cytokine levels. In accordance with these results, a previous study by Hafez et al. 2020 reported these anti-inflammatory, antioxidant, and anti-apoptotic actions of RU in an animal model of diabetic nephropathy. The present study offers evidence that PAF is involved in the pathogenesis of 5-FU-associated hepatotoxicity in rats with a potential ameliorative action of RU.

## Conclusion

In conclusion, the administration of 5-FU exhibited a severe liver damage that was confirmed biochemically and histopathologically. PAF is involved in the pathogenesis of 5-FU-associated hepatotoxicity. Furthermore, the pretreatment with RU attenuated 5-FU-induced hepatotoxicity. The hepatoprotective potential of RU can be attributed to its anti-inflammatory, anti-apoptotic, and antioxidant properties mediated by antagonizing PAF receptors, attenuating the PAF-mediated response, and up-regulating HO-1 production. Thus, the use of RU might prove beneficial for the well-being of the cancer patients. Future studies should unravel other mechanisms of action of RU, study the dose-dependent effect, optimize the correct dose for human use, and compare the drug's efficacy and safety with other suggested therapies.

## Funding sources

This research did not receive any specific grant from funding agencies in the public, commercial, or not-for-profit sectors.

## Ethical approval

The experimental animal study was performed following ARRIVE ethical guidelines and approved by the board of the Faculty of Medicine, Minia University, Egypt (239:7/2019).

## Competing interests

The authors declare no competing interests.

## Data Availability

Data are available with the corresponding author upon reasonable request.
